# Iron Deficiency Anemia, Not Iron Deficiency, Is Associated with Reduced Attention in Healthy Young Women

**DOI:** 10.3390/nu9111216

**Published:** 2017-11-05

**Authors:** Rebecca L. Cook, Nicholas J. O’Dwyer, Helen M. Parker, Cheyne E. Donges, Hoi Lun Cheng, Katharine S. Steinbeck, Eka P. Cox, Janet L. Franklin, Manohar L. Garg, Kieron B. Rooney, Helen T. O’Connor

**Affiliations:** 1 Faculty of Health Sciences, Discipline of Exercise and Sport Science, The University of Sydney, Lidcombe, NSW 2141, Australia; rebecca.cook@sydney.edu.au (R.L.C.); nicholas.odwyer@sydney.edu.au (N.J.O.); ecox8840@uni.sydney.edu.au (E.P.C.); kieron.rooney@sydney.edu.au (K.B.R.); helen.oconnor@sydney.edu.au (H.T.O.); 2 School of Exercise Science, Sport and Health, Charles Sturt University, Bathurst, NSW 2795, Australia; cheyne_donges@outlook.com; 3 Charles Perkins Centre, The University of Sydney, Camperdown, NSW 2006, Australia; helen.cheng@health.nsw.edu.au; 4 Academic Department of Adolescent Medicine, The Children’s Hospital at Westmead, Westmead, NSW 2145, Australia; kate.steinbeck@health.nsw.gov.au; 5 Sydney Medical School, Discipline of Child and Adolescent Health, The University of Sydney, Westmead, NSW 2145, Australia; 6 Metabolism and Obesity Services, Royal Prince Alfred Hospital, Camperdown, NSW 2050, Australia; janet.franklin@sswahs.nsw.gov.au; 7 School of Biochemical Sciences and Pharmacy, University of Newcastle, Callaghan, NSW 2308, Australia; manohar.garg@newcastle.edu.au

**Keywords:** iron deficiency, cognition, attention, anemia, young adults, young women

## Abstract

Women of reproductive age are at increased risk for iron deficiency (ID) and iron deficiency anemia (IDA), with both implicated in decreased cognitive function (CF). Obesity may complicate this association via inflammatory-mediated ferritin elevation. This cross-sectional study examined the association between hematological iron status (iron replete (IR), ID or IDA) and CF in healthy, young (18–35 years) women of normal-weight (NW: BMI 18.5– 24.9 kg/m^2^) or obese-weight (OB: BMI >30 kg/m^2^). Participants completed a validated, computer-based cognition assessment evaluating impulsivity, attention, information processing, memory and executive function; CF reported as *z*-scores (mean ± SD). Iron status and CF were compared between groups via ANOVA, with adjustment for potential confounders (BMI, physical activity, C-reactive protein) via ANCOVA. A total of 157 NW and 142 OB women (25.8 ± 5.1 years) participated. Prevalence of ID and IDA were 14% and 6% respectively, with no significant difference between NW and OB groups. Women with IDA scored significantly lower on attention (although within normal range; ±1 *z*-score), compared to ID (IDA: −0.75 ± 1.89; ID: 0.53 ± 1.37; *p* = 0.004) but not IR (0.03 ± 1.33, *p* = 0.21) groups; there were no significant differences between ID and IR groups (*p* = 0.34). Adjustment for confounders did not significantly alter these results. In conclusion, women with IDA showed significantly reduced attention compared to women with ID.

## 1. Introduction

Iron is an essential nutrient and has a number of key roles in the body including erythropoiesis and oxygen transportation, energy production, enzyme synthesis, and immune function [1]. Iron is also known to play a crucial role in brain development and maintenance of neuronal activity and networks [2,3]. Iron deficiency (ID), including its more severe form iron deficiency anemia (IDA), is the most prevalent single nutrient deficiency worldwide, affecting both developed and developing countries [4]. Women of reproductive age are particularly at risk due to increased iron requirements secondary to menstruation [5,6].

Current literature linking ID and suboptimal cognitive function is heavily weighted towards studies in infants and children [3,7,8,9]. However, a recent (2013) systematic review in women of reproductive age [10] examined the relationship between iron status and cognitive function, and additionally, whether a change in iron status affects cognitive performance via iron supplementation. There was variation in the hematological methods used to assess iron status, and the severity of iron deficiency was not uniformly reported by all studies. Lower cognitive performance was reported in those with IDA or ID compared to iron replete (IR) women in four out of eight studies, across various psychometric tasks measuring attention, memory, learning, intelligence and arithmetic scores. Seven out of 10 studies reported improved cognition after iron supplementation intervention. Meta-analysis (with only three of the 10 studies having sufficient data, only one of which reported participants with IDA) found significant improvement for arithmetic scores (*p* < 0.001), whereas changes in attention, working memory and visuo-spatial ability were non-significant [10].

This abovementioned review identified limitations of existing studies including large study heterogeneity, small sample sizes, non-validated measures of cognitive assessment, varying classifications of iron status, poor study quality and lack of confounder adjustment [10]. Major confounders which current studies rarely address are obesity and its co-morbidities. Obesity-related systemic inflammation is associated with reduced cognition [11], and in addition, has known links to ID [12,13], mediated by elevated levels of hepcidin—an iron regulatory hormone that controls gut iron absorption and movement of iron stores within the body [14]. Thus, low iron status secondary to obesity-related inflammation may, in turn, deleteriously affect cognition in young women. Evidence also indicates that metabolic disease and physical inactivity are associated with both obesity and reduced cognition [11,15,16,17].

As ID is the most prevalent deficiency in young women of reproductive age and there is inconclusive evidence regarding its impact on cognitive function, the primary aim of this study was to examine the association between ID and cognitive function in this population. A secondary aim was to examine the relationship between cognitive function and IDA—a more serious deficiency which affects a smaller proportion of young women. Our hypothesis was that women with ID would show significantly lower cognition scores compared to women with IR. Cognitive impairment was also expected to be greater for those with IDA. This study also addressed the limitations of current research by adjusting the analyses for the potential confounding effects of obesity, systemic inflammation and physical activity. 

## 2. Materials and Methods

A cross-sectional study (“Food, Mood & Mind Study”) was conducted on a convenience sample of normal weight (NW) (BMI: 18.5–24.9 kg/m^2^) and obese weight (OB) (BMI: ≥30.0 kg/m^2^) young women aged 18–35 years. The upper age limit of 35 years was used to reduce confounding from age-related cognitive decline [18].

To facilitate examination of the potential impact of obesity and associated systemic inflammation, 50% (*n* = 150) of the 300 participants targeted for recruitment were to have obesity. Women with obesity rather than overweight were targeted so as to establish a substantial BMI gap between the NW and OB group and maximize the capacity to observe significant weight-related differences. Participants were recruited from metropolitan (Sydney) and regional/rural (Bathurst) areas within the state of New South Wales in Australia. To ensure adequate representation of rural participants, one third (*n* = 100; 50% NW, 50% OB) of the participants were recruited from the regional/rural site. 

A range of methods were used to recruit participants including flyers, websites, newspaper advertisements, e-newsletters, social media, radio and letterbox drops. This combination of active and passive recruitment strategies is recommended as best for optimizing recruitment of young adults for research [19]. The study was approved by Human Research Ethics Committees linked to local health district services and participating universities (protocol numbers: X10-0259, HREC/10/RPAH/455 and 2014/050). Written informed consent was obtained from all participants prior to study commencement. Participants who completed the study received a gift card (AUD$100) to cover time and travel costs.

Volunteers were screened for eligibility prior to their first study visit. Participants were eligible if they were healthy, with a BMI in the normal weight (18.5–24.9 kg/m^2^) or obese (≥30.0 kg/m^2^) category according to the WHO guidelines [20]. Exclusion criteria included significant medical conditions (e.g., cardiovascular or metabolic disease including type 2 diabetes) and conditions which may compromise: (1) cognitive function including neurological or psychiatric conditions, use of medications/substances known to alter mood, reaction time or cognitive capacity including smoking, alcohol consumption ≥50 g per week and recreational drug use; (2) ability to complete the touch-screen cognition testing including vision, hearing, or motor coordination problems and English literacy; (3) iron status including pregnancy, breastfeeding, regular blood donations (three or more donations per year or having donated blood in the previous three months). Volunteers receiving treatment for known iron deficiency and those taking iron supplements for general health were also excluded.

Participants attended two study visits, at a University research laboratory or an obesity clinic of a major teaching hospital, approximately one week apart. The first session involved informed consent, anthropometric measurement and cognitive assessment. All cognitive assessments were conducted after breakfast and prior to 1300 h. Participants were asked to refrain from heavy exercise, alcohol and caffeine (and other stimulants) on the morning of the cognition test and to consume their usual breakfast to ensure they felt comfortable and not hungry during the testing session. Caffeine was excluded as it has been shown to reduce fatigue and help increase attention. This may mask subtle cognitive deficits resulting from ID or IDA [21]. The second visit involved collection of a fasting blood sample. 

Anthropometric measurements were taken in light clothing (no shoes) at the initial visit. Height was measured to the nearest 0.1 cm in duplicate with a portable stadiometer (Seca 213, Hamburg, Germany). Body weight was recorded on a digital platform scale accurate to 0.1 kg (PW-200KGL; A&D Weighing, Thebarton, SA, Australia). Waist circumference was measured at the midpoint between the lowest rib and iliac crest to the nearest 0.1 cm in duplicate (mean reported) with a retractable metal tape (Lufkin W606PM; Cooper Industries, Sparks, NV, USA) according to International Diabetes Federation Guidelines [20].

Cognitive function was also assessed at the initial visit, using a validated computer-based cognitive assessment battery (IntegNeuro^TM^; Brain Resource, Woolloomooloo, NSW, Australia) via a touch-screen and headset. The duration of the assessment was one hour. This platform has well-established validity, reliability, cross-cultural consistency and norms [22,23]. The five cognitive domains of impulsivity, attention, information processing, memory and executive function were assessed using the following tests: the Go-NoGo task (impulsivity); the continuous performance task (attention); the switching of attention test (trail making part B) and a choice reaction time task (information processing); the memory recognition (immediate and delayed) task and the digit span (forward and reverse) test (memory); the maze test (executive function). Results are reported as *z*-scores, adjusted for age and years of education using internal regression methods based on Brain Resource’s extensive normative databank [24], with this adjustment occurring prior to statistical analysis. *Z*-scores between ±1 are within normal range, with positive and negative scores reflecting above- and below-average performance, respectively. Participants completed the test using their dominant hand seated in a quiet, appropriately lit, air-conditioned location. Phase of menstrual cycle was not standardized as recent evidence indicates that its effect is small and not clinically significant [25,26,27].

The second study visit involved a morning, fasted (12 h) venous blood draw (within 14 days of cognitive testing). Biochemical analyses included routine iron studies (serum ferritin, serum transferrin, transferrin saturation), serum transferrin receptor (sTfR), hemoglobin (Hb) (from whole blood) and markers of inflammation (serum C-reactive protein, CRP; and serum alpha-1-acid glycoprotein; α1GP). Iron markers and CRP were analyzed via automated immunoassay and rate nephelometry, respectively, on the Roche COBAS 8000 e602. Hemoglobin was measured via absorption spectrophotometry and flow cytometry on the Abbot CELL-DYN Sapphire System. α1GP was analyzed using immunoturbidimetry on Konelab™ 20XT Clinical Chemistry Analyzer (using Thermo Fisher reagents). All biochemical analyses were performed by a NATA accredited diagnostic laboratory. Full blood count was collected and analyzed within 6 h by the hospital laboratory. All of the other samples were collected, placed in ice and then spun at 3000 RPM (2123 g-force) at 4 °C for 10 min.

Participants were classified as ID if ferritin was <20 μg/L (reference range: 20–300 μg/L) and IDA if Hb was <120 g/L (reference range: 120–150 g/L) [6]. CRP and/or α1GP levels above the reference range (CRP > 5.0 mg/L; α1GP > 1.0 g/L) were indicative of elevated inflammation [28]. Inflammation is known to increase ferritin levels and may lead to misclassification (missed cases) of ID. Thus, correction factors were applied to the raw ferritin values and re-classified against the laboratory-specific reference range [28]. The correction factors used were as follows: 0.77 (raised CRP only), 0.75 (raised α1GP only) and 0.53 (raised CRP and α1GP) [28]. Both the raw and corrected ferritin values were used in the analysis against cognitive performance. Total Body Iron was calculated from serum ferritin (µg/L) and sTFR (mg/L) using the following equation: total body iron (mg/kg) = −(log10 (sTFR × 1000/ferritin) − 2.8229)/0.1207 [28,29]. Moreover, to enable comparison with recently published data from a similar population [30], iron status was also classified using the four-variable marker model of iron status (ID present when ≥2 of the following four thresholds were met: ferritin < 20 μg/L, sTfR > 2.3 mg/L, transferrin saturation < 16%, red cell distribution width ≥15%; using cut-off values relevant to the laboratory reference ranges in the present study) [30].

The short form of the International Physical Activity Questionnaire (IPAQ-S) was used to assess overall physical activity (PA) in Metabolic Equivalent of Task (MET) minutes per week. The IPAQ-S assesses duration (minutes) and frequency (days) of walking, moderate-intensity and vigorous-intensity activities per week and sitting hours per weekday.

The sample size required to detect significant cognitive differences between ID and IR groups was estimated from the study of Murray-Kolb et al. [31] which reported statistically significant differences between a sample of 73 ID women and 43 IR controls. Prevalence of ID was estimated using a study in young women (18–35 years) attending an Australian university which reported 33% ID [32] and a large lifestyle study (25–49 years) which reported a prevalence of ~20% [33]. Taking 25% as an intermediate estimate of prevalence, a total sample of approximately 300 was required to provide 75 participants with ID.

Unpaired *t*-tests were used to compare the NW and OB groups on demographic characteristics, and iron and inflammatory status. Chi-squared analyses were conducted for iron, CRP, PA and BMI. Analysis of the relationship between iron status and cognitive function was investigated using two separate models via analysis of variance (ANOVA). The data were checked for normality prior to running the models; outliers with *z*-scores greater than ±4 were removed prior to analysis (<4%, *n* = 11) as these data were considered to be spurious. The first model was constructed using a 3 × 5 ANOVA with iron status as the independent grouping variable (IR vs. ID vs. IDA) and the five cognition *z*-scores (impulsivity, attention, information processing, memory, executive function) as repeated measures factors (since all participants were measured on the five cognitive domains in the assessment battery). Tukey’s post-hoc tests were used in all cases to locate any significant differences observed. Analysis of covariance (ANCOVA) models were constructed using CRP, PA and BMI as covariates. The covariates were applied individually as well as concurrently, since there was some correlation between them (BMI-CRP: *r* = 0.67, *p* < 0.0001; BMI-PA: *r* = −0.22, *p* < 0.001; CRP-PA: *r* = −0.14, *p* < 0.05), thereby introducing a degree of redundancy when they were applied concurrently. 

Univariate analyses were carried out within each ANOVA and ANCOVA model to examine between-group differences in *z*-scores for each cognitive domain. There were no repeated measures factors in these analyses. Non-parametric tests (Kruskal–Wallis one-way ANOVA) also were applied as appropriate. Significance for all analyses was set at *p* < 0.05. Results are reported as mean ± SD. All statistical analyses were carried out using Statistica software (v.12; StatSoft Inc., Tulsa, OK, USA).

The number of participants at the metropolitan location was 200 (67%) and at the regional location was 99 (33%). All of the tests above also were applied to the two groups based on study location, in order determine whether there were any systematic effects on demographic characteristics, iron or inflammatory status, or cognitive function.

## 3. Results

### 3.1. Participant Characteristics

**Figure 1:**
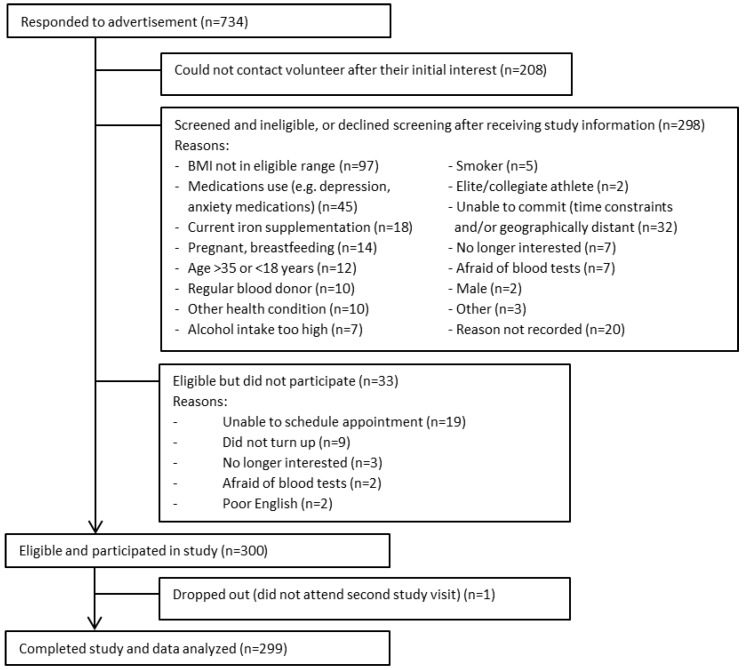
Participant recruitment and study participant flow chart.

A total of 300 women were recruited to the study. All except one completed both study visits (NW: *n* = 157; OB: *n* = 142); complete data (blood, cognition results, BMI, PA and CRP) were available for 285 participants. All participants completing the study confirmed compliance to pre-testing conditions. The flow of participant recruitment is summarized in Figure 1. Major reasons for exclusion included: BMI outside of study limits (32%); depression or anxiety medications (15%); time constraints or geographical location (11%); current iron supplementation (6%); refusal of blood testing (5%); pregnancy or breastfeeding (5%); and age outside of study limits (4%).

Participant characteristics are summarized in Table 1. Average age was 25.8 ± 5.1 years, with OB women significantly older by approximately 2 years compared to NW women (*p* < 0.001). Overall mean years of education was 16.2 ± 2.2 years, with NW women reporting slightly but significantly longer duration of education than OB women. Over half of the participants had completed university education. The distribution of the highest qualification was significantly different between groups (*p* = 0.018), with more completing university in the NW and more completing technical college in the OB group.

The mean BMI of the OB group was significantly higher than the NW group (*p* < 0.001). Most (55%) of the OB women had BMIs consistent with Obese Class I (30.0–34.9 kg/m^2^), with lower proportions classified as Class II (BMI 35.0–39.9 kg/m^2^) and Class III (BMI 40.0 kg/m^2^). Waist circumference in 97% of the OB participants was above the recommended guidelines (80 cm) for reducing risk of metabolic disorders and cardiovascular disease [20]. Furthermore, 85% of OB women had a waist measure above the threshold for very high risk of chronic disease (>88 cm) [20]. Total PA levels were significantly lower in the OB group (*p* < 0.001).

### 3.2. Iron and Inflammatory Status

Using the raw ferritin values, ID and IDA prevalence was 14% (*n* = 41) and 6% (*n* = 18), respectively. The proportion of participants with ID and IDA was not significantly different between the NW and OB groups (Table 2). After correction for inflammation, the percentage of women with ferritin levels consistent with ID increased to 20% (*n* = 60), with more in the OB than the NW group (NW: 14%; OB: 27%; *p* = 0.010). Total body iron was similar between NW and OB groups (Table 2) using both the raw and corrected serum ferritin levels. Comparison of inflammatory markers showed significantly higher mean CRP and α1GP levels in the OB group. Furthermore, a larger proportion of OB women showed elevated CRP and/or α1GP levels (Table 2).

### 3.3. Iron Status and Cognition

**Figure 2:**
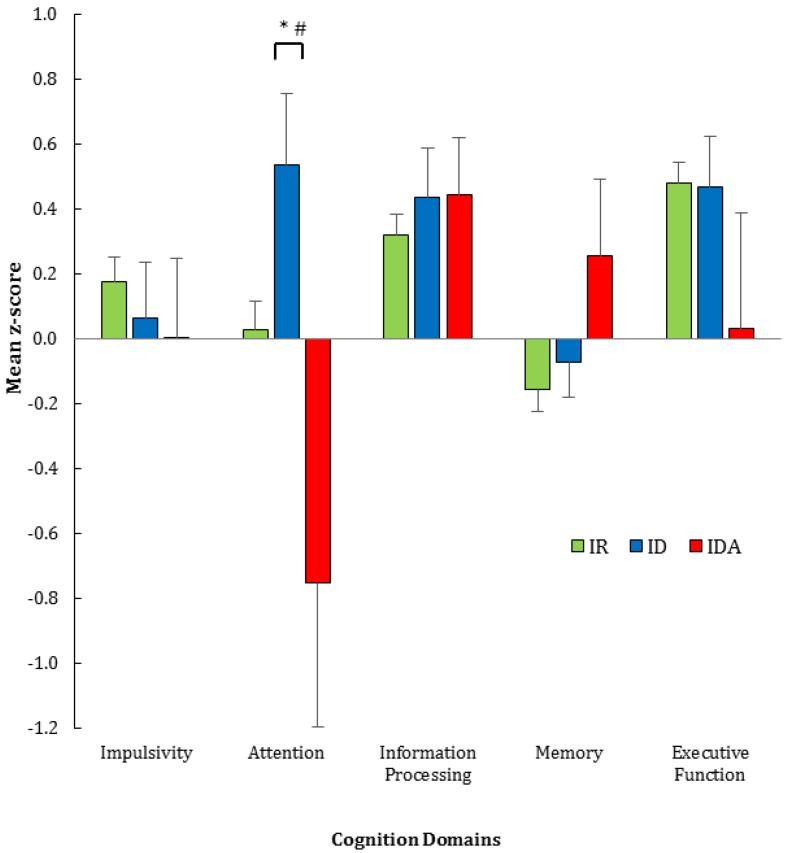
Iron status (IDA, *n* = 225; ID, *n* = 42; IR, *n* = 18) vs. performance in five cognitive domains. Mean *z*-score ± SE (standard error); normal *z*-score is +1 to −1. * Denotes *p* < 0.01 on post hoc tests: IDA vs. ID; ^#^ denotes *p* < 0.01 on univariate analysis IDA vs. ID. Abbreviations: IDA, iron deficiency anemia (hemoglobin < 120 g/L); ID, iron deficiency (ferritin below normal range, but hemoglobin ≥ 120 g/L); IR, iron replete. Domain scores adjusted for age and education. Attention was measured using a computerized continuous performance task, which tests the ability to maintain attention and inhibit impulsive responding over time [24].

The cognitive function of the three groups based on iron status (IR, ID and IDA) was assessed across five domains: impulsivity, attention, information processing, memory and executive function. Complete data (blood, cognition, and confounders PA, BMI and CRP) were available and analyzed for 285 participants; of these, 80% were classified as IR, 14% ID and 6% IDA. Mean *z*-scores for each cognitive domain were within normal range (±1 *z*-score) for all iron status groups (Figure 2), with little difference between groups except in the attention domain. The *z*-scores for ID were similar to the IR group. Repeated measures ANOVA showed no overall group difference but there was a significant group × domain interaction (*p* < 0.01). Post-hoc analyses showed a significantly lower *z*-score on the attention domain in the IDA compared to ID group (*p* = 0.004) but not the IR group. Univariate analyses of the three groups also showed a significant effect for attention (*p* = 0.005). In light of the differences between groups in the standard errors for attention (Figure 2), non-parametric tests also were applied and confirmed the differences shown by the parametric tests, with IDA lower than ID (*p* = 0.012) but not IR.

### 3.4. Influence of Covariates on Iron Status and Cognition

Analysis of covariance was conducted to determine the potential influence of BMI, chronic inflammation (CRP) and PA (total MET-min/week) on cognitive function. The three covariates were similar between the three iron status groups (BMI: *p* = 0.074; CRP: *p* = 0.17; PA: *p* = 0.28). 

The effect of each covariate on cognitive performance was first examined individually in separate ANCOVA models and then all three covariates were included in a single model to assess their overall effect on cognition. There were no overall group effects (*p* ≥ 0.18), but significant group × cognitive domain interactions were seen after adjusting for BMI (*p* < 0.001), CRP (*p* < 0.01) and PA (*p* < 0.01) separately and combined (*p* < 0.001). Post-hoc tests again showed significant differences between ID and IDA groups in the attention domain, after individual adjustment for each of the three covariates (BMI: *p* = 0.004; CRP: *p* = 0.004; IPAQ: *p* = 0.004) and combined adjustment (*p* = 0.004), but not between the IDA and IR (*p* ≥ 0.20) or ID and IR (*p* ≥ 0.32) groups. Univariate analysis of each cognitive domain across the three groups was significant in the attention domain after individual adjustment for each covariate (BMI: *p* = 0.002; CRP: *p* = 0.006; IPAQ: *p* = 0.005) and combined adjustment of all covariates in one ANCOVA model (*p* = 0.001). Therefore, the ANCOVAs did not change the overall finding of a significantly lower (but still in the normal range) attention domain *z*-score for the IDA group when compared with the ID group, but not compared to the IR group.

### 3.5. Corrected Ferritin Analysis

Reclassification of ID based on inflammatory status added 19 more participants with ID and yielded a similar pattern of results to the unadjusted ID data. Repeated measures ANOVA again showed no significant overall group difference across the five cognition domains (*p* = 0.54) and a significant group × domain interaction (*p* < 0.01), but the post-hoc test on the attention domain was now borderline (*p* = 0.063). The univariate test was still significant for IDA vs. ID on the attention domain (*p* = 0.03), as was the nonparametric test (*p* = 0.045). All ANCOVAs (using BMI, CRP, PA, individually and combined) showed precisely this same pattern of results.

### 3.6. Four-Variable Marker Model of Iron Status

Using the four-variable marker model resulted in the classification of 33 participants as ID without correction for inflammation and 42 after correction for inflammation (IDA was not defined by this method) [30]. Repeated measures analyses indicated no differences between ID and IR groups for any cognitive domain, regardless of ferritin-correction or adjustment for confounders (all *p* > 0.05).

### 3.7. Participant Location

Participants from metropolitan and regional locations were similar in age (*p* = 0.59) and BMI (*p* = 0.44). Their iron status also was similar, whether based on raw (*p* = 0.29) or corrected (*p* = 0.27) ferritin levels. Cognitive performance across all domains was equivalent in metropolitan and regional participants (*p* = 0.41).

## 4. Discussion and Conclusions

This cross-sectional study examined the association between iron status and cognitive function in young NW and OB women. While cognitive function of the participants was within normal range, women with IDA showed significantly lower performance in the attention domain, even after adjustment for potential confounding from BMI, inflammation and physical activity. Women with ID did not perform differently to those who were IR, suggesting cognition does not decline significantly with stage 1 and 2 (non-anemic) ID. Of the three confounders, BMI appeared to be most strongly linked to cognition. As only a small proportion of the participants had IDA (6%), a small decrease in attention even when within the normal range deserves further examination in a larger sample.

The prevalence of ID (14%) and IDA (6%) in this study is broadly similar to figures reported in the general adult (20–50 years) Australian female population. The AusDiab study of 1634 Australian adults found that of the 444 women aged 25–49 years, 20% and 4% were reported as having ID and IDA respectively [33]. However, in a similar sample of young (18–35 years) women in a university setting, ID prevalence was reported to be ~33% [32]. The lower prevalence of ID in the current study may be partly attributed to exclusion of regular blood donors and those with chronic iron deficiency. Interestingly, correction of ferritin levels for inflammation resulted in a substantial increase in apparent ID from 14% to 20%, with no differences observed in the proportion of participants with ID or IDA in relation to BMI. 

Recent reviews have identified an association between iron status and cognitive performance in young women [10,34,35] and an association with academic performance has also been reported [36]. Empirical studies have reported changes in cognitive performance associated with changes in iron status [31,34,37,38,39]. The cognitive domain of attention was frequently identified [30,31,34,35,36,37,38,39], consistent with the finding in the present study that women with IDA had significantly lower attention scores than those with ID. No differences were found here between the IR and ID groups. Other studies have found positive associations between iron status and information processing [37] and planning ability in women with and without anemia [30], and interestingly, a negative association with working memory [30] which was not observed in the current study. However BMI and other confounders were not controlled in those analyses.

Possible mechanisms of action for the relationship between iron status and cognition are still being explored. Hypotheses include inadequate brain iron availability, which affects neurotransmission and signaling, myelination, neurometabolism, and gene profiles [40,41,42]. Rat models indicate that adequate serum iron maintains brain iron [43] and that iron deficiency leads to diminished central dopaminergic transmission and receptor trafficking, with the D_2_ receptor particularly affected [43,44,45]. Dopamine may be directly involved in regulating attention [42], which appears to depend on a distributed large-scale network mediated by the dorsolateral prefrontal and mesial frontal cortex, thalamus, basal ganglia and posterior parietal and superior temporal lobes [46]. Our results support the need for additional research, particularly in IDA. The use of electroencephalogram (EEG) and imaging techniques such as functional magnetic resonance imaging (fMRI), in combination with psychometric assessment, may help to identify structural and functional areas of the brain that may be involved in the pathogenesis of reduced cognitive performance, particularly attention, associated with IDA.

Major strengths of this study include recruitment of a well-educated healthy cohort, free of comorbidities and with exclusion of potential confounders (smoking, excess alcohol, diagnosis or recent history of mental health disorders and chronic disease). This study is one of the first to comprehensively account for a number of key confounding variables when examining the influence of iron status on cognitive function in young women. 

One of the major challenges in the cognitive testing literature is the diversity and classification of tests. Similar tests are classified under different domains, many tests measure multiple constructs, and there is a wide range of tests to evaluate specific cognitive domains. There is currently no consensus regarding the classification of cognitive tests and domains [47], which may explain some of the discrepancies between studies. Attention and impulsivity measures are often classified under the executive function domain, and working memory (as it measures multiple constructs) has been reported across a range of domains in the literature including attention, information processing, memory and executive function [24,47]. In the current study, attention was assessed using a continuous performance test which measures sustained attention and the capacity to inhibit impulsive responding over time [24]. It is plausible that IDA specifically reduces the capacity to sustain attention as it is well known to be associated with fatigue [48]. 

The study was primarily powered to detect changes in cognitive function with ID and the number of participants recruited with IDA was small, which presents an important limitation in examining the association with cognitive performance. However, even with a small sample, significant relationships were observed for IDA in the attention domain which warrants further study in a randomized trial design. Although we used the published four-variable marker model of iron status [30] to further confirm our findings, the laboratory norms relevant to our iron assays (rather than those appropriate to the assays in the published work) were applied. The differences in normative values may have limited our capacity to observe significant differences. Self-reported measures for PA were used to reduce the respondent burden, but these have recognized limitations. In addition, although BMI is not a precise measure of adiposity, the waist circumference of the women with obesity indicates that 97% of this sample had central fat distribution indicative of excess body fat (Table 1). Based on existing evidence we did not standardize measurement for phase of menstrual cycle, however it is possible that menstrual cycle influences cognitive function in women of reproductive age. 

This study found decreased cognitive performance with IDA in young healthy women, however no significant associations with reduced cognitive function were observed for ID. Although IDA was associated with reduced attention, performance was still within the normal range. The clinical or functional significance of these findings deserves further research, potentially with EEG or imaging, in addition to cognition testing pre- and post-intervention to rectify deficiency. Assessment of genetic factors, particularly dopamine receptor genes that have been implicated in iron and cognition status [41,42], may help to delineate these relationships further.

## Figures and Tables

**Table 1:** Demographic characteristics of participants.

	All Participants (*n* = 299)	NW Group (*n* = 157)	OB Group (*n* = 142)	*p*-Value *
Age (years)	25.8 ± 5.1	24.9 ± 4.6	26.9 ± 5.4	<0.001
Education (years)	16.2 ± 2.2	16.5 ± 2.2	15.9 ± 2.2	0.022
Highest qualification: (*n*, %)				0.018
Secondary school	82 (28%)	43 (28%)	39 (27%)	
Technical college	47 (16%)	16 (10%)	31 (22%)	
University	169 (57%)	97 (62%)	72 (51%)	
Height (cm)	165.4 ± 6.9	165.5 ± 7.3	165.3 ± 6.6	0.805
Weight (kg)	78.1 ± 23.5	59.7 ± 7.0	98.5 ± 17.9	<0.001
BMI (kg/m^2^)	28.6 ± 8.6	21.8 ± 1.7	36.1 ± 6.8	<0.001
Obese Class I	79 (26%)	N/A	79 (56%)	
Obese Class II	41 (14%)	N/A	41 (29%)	
Obese Class III	22 (7%)	N/A	22 (15%)	
Waist circumference (cm)	84.5 ± 18.7	69.7 ± 4.2	101.2 ± 14.1	<0.001
Below 80 cm (*n*, %)	156 (52%)	154 (98%)	2 (1%)	<0.001
80–88 cm inclusive (*n*, %)	20 (7%)	3 (2%)	17 (12%)	
Above 88 cm (*n*, %)	120 (40%)	0 (0%)	120 (85%)	
Physical activity (MET-min/week)	2603 ± 2141	3076 ± 2302	2080 ± 1815	<0.001

Data reported as mean ± SD (unless otherwise specified). * *p*-Value for continuous variables: unpaired *t*-test, NW vs. OB. *p*-Value for categorical variables: Chi-square test, NW vs. OB. Data missing for waist circumference (OB: *n* = 3). Obese Class I = 30.0–34.9 kg/m^2^; Obese Class II = 35.0–39.9 kg/m^2^; Obese Class III ≥ 40.0 kg/m^2^. BMI, body mass index; MET, metabolic equivalent of task; min, minute; N/A, not applicable.

**Table 2:** Participant iron and inflammatory status.

	All Participants (*n* = 299)	NW Group (*n* = 157)	OB group (*n* = 142)	*p*-Value *
Iron Studies				
Serum ferritin (µg/L)	49.9 ± 38.3	47.3 ± 31.4	52.7 ± 44.7	0.22
Hb (g/L)	134.1 ± 9.9	132.9 ± 9.7	135.4 ± 10.1	0.035
sTfR (nmol/L)	1.2 ± 0.4	1.1 ± 0.3	1.3 ± 0.4	<0.001
Transferrin saturation (%)	24.5 ± 10.4	27.1 ± 10.8	21.6 ± 9.2	0.103
RCDW (%)	12.5 ± 1.1	12.3 ± 1.1	12.7 ± 1.1	0.461
IDA (*n*, %)	18 (6%)	13 (8%)	5 (4%)	0.21
ID (*n*, %)	41 (14%)	21 (14%)	20 (14%)	
Replete (*n*, %)	235 (80%)	119 (78%)	116 (82%)	
Total body iron (mg/kg)	10.7 (3.1)	10.6 (3.0)	10.8 (3.3)	0.489
Corrected serum ferritin ** (µg/L)	42.4 ± 32.0	45.3 ± 30.6	39.4 ± 33.4	0.11
ID (*n*, %)	60 (20%)	22 (14%)	38 (27%)	0.010
Replete (*n*, %)	216 (74%)	118 (77%)	98 (70%)	
Total body iron (mg/kg)	10.4 (3.1)	10.6 (3.0)	10.2 (3.3)	0.256
CRP (mg/L)	3.4 ± 4.3	1.4 ± 2.1	5.5 ± 5.0	<0.001
CRP < 5 mg/L	233 (78%)	149 (95%)	84 (59%)	<0.001
CRP ≥ 5 mg/L	63 (21%)	6 (4%)	58 (41%)	
α1GP (mg/L)	0.75 ± 0.22	0.60 ± 0.14	0.90 ± 0.19	<0.001
α1GP < 1 mg/L	257 (86%)	153 (98%)	104 (73%)	<0.001
α1GP ≥ 1 mg/L	37 (12%)	0 (0%)	37 (26%)	
Summary of inflammatory status:				
Normal CRP + normal α1GP	218 (73%)	147 (96%)	71 (50%)	<0.001
Raised CRP only	39 (13%)	6 (4%)	33 (23%)	
Raised CRP + α1GP	24 (8%)	0 (0%)	24 (17%)	
Raised α1GP only	13 (5%)	0 (0%)	13 (9%)	

Data reported as mean ± SD. * *p*-Value for continuous variables: unpaired *t*-test, NW vs. OB. *p*-Value for categorical variables: Chi-square test, NW vs. OB. ** Ferritin corrected for inflammatory status (CRP ≥ 5 mg/L; α1GP > 1.0 mg/L) [28]. Missing data: iron studies (ferritin, NW *n* = 3; OB *n* = 1), α1GP (NW *n* = 4, OB *n* = 1). Normal biochemical reference ranges: serum ferritin 20–300 µg/L; Hb > 120 g/L; CRP < 5 mg/L; sTFR 0.9–2.3 mg/L; α1GP < 1 mg/L. Total body iron calculated from serum ferritin and sTFR [28,29]. α1GP, alpha-1-acid glycoprotein; CRP, C-reactive protein; Hb, hemoglobin; IDA, iron deficiency anemia; ID, iron deficiency; NW, normal weight; OB, obese weight; RCDW, red cell distribution width; sTfR, serum transferrin receptor.
